# Investing for sustainable health and well-being: bringing the Helsinki commitment to life

**DOI:** 10.1093/eurpub/ckag033

**Published:** 2026-04-03

**Authors:** Tit Albreht, Charlotte Marchandise

**Affiliations:** EUPHA President; Executive Director

At the European Public Health Conference in Helsinki, we collectively reaffirmed a simple but powerful commitment: health and well-being must be placed at the centre of political, economic, and societal decisions. The Helsinki Statement reminded us that sustainable societies are built when policies align with health, equity is prioritized, democratic governance is strengthened, trust is protected, and the workforce is supported.

European Public Health Week 2026 is our opportunity to make that commitment visible in practice.

The theme ‘*Investing for sustainable health and well-being*’ reflects a growing body of evidence that the strongest determinants of health lie beyond healthcare systems, in housing, education, employment, climate, digital environments, and governance structures. Investment in health is therefore not a sectoral expenditure; it is a cross-government and cross-society strategy.

Throughout the week, we will explore five interlinked dimensions of this investment.

**First, aligning all policies with public health.** 

Health in All Policies and Health for All Policies are no longer theoretical frameworks. Across Europe, municipalities integrate health equity into climate adaptation, transport systems prioritize walkability, and planning decisions incorporate health impact assessments. These incremental practices demonstrate that alignment is possible when co-benefits are recognized and governance mechanisms enable collaboration.

**Second, prioritizing equity through inclusive investment.** 

Health inequities remain systematic, socially determined, and avoidable. Equity-orientated investment requires moving beyond universal approaches alone and addressing structural barriers. Inclusive budgeting, culturally sensitive service design, participatory governance, and data systems that monitor inequities are practical tools for ensuring that no one is left behind. Equity is not an additional cost; it is a stabilizing investment in social cohesion and resilience.

**Third, protecting health through democratic governance and accountability.** 

Governance is itself a determinant of health. Transparent decision-making, meaningful participation, and accountable institutions build trust and strengthen policy coherence. As public institutions face increasing scrutiny and complexity, investing in democratic processes is essential for sustaining legitimate and effective health systems.

**Fourth, countering disinformation with trust and transparency.** 

Public health depends on trust. In an era shaped by algorithmic information environments and large language models, misinformation cannot be addressed by correction alone. Trust grows when institutions listen, explain trade-offs openly, acknowledge uncertainty, and engage communities respectfully. Strengthening relational practice, communication integrity, and accountability is therefore a core public health investment.

**Fifth, strengthening and supporting the health workforce.** 

A resilient and valued workforce underpins all other reforms. Europe faces persistent workforce shortages, demographic shifts, and growing complexity of care. Sustainable responses require long-term planning, supportive working environments, intersectoral governance, and meaningful engagement of professionals in shaping reform. Investment in people is investment in system sustainability.

These five themes are not separate silos. They form a coherent architecture for sustainable well-being. Alignment enables equity. Equity requires participation. Participation builds trust. Trust strengthens institutions. Institutions support the workforce. The workforce delivers health.

European Public Health Week is not simply a calendar event. It is a collective mobilization across countries, sectors, and disciplines. Local authorities, national public health associations, academic institutions, civil society organizations, youth networks, and European partners all contribute to making health visible in policy debates and community action.

In 2026, we invite you to move from principle to practice. Share examples of intersectoral collaboration. Highlight inclusive investment strategies. Showcase democratic participation initiatives. Demonstrate how trust is strengthened in your community. Present innovative workforce solutions.

By doing so, we give substance to the Helsinki commitment and demonstrate that investing in sustainable health and well-being is both feasible and necessary.

Health is not the outcome of one ministry, one programme, or one profession. It is the result of collective efforts. European Public Health Week 2026 is our shared space to make those choices visible.

Conflict of interest: None declared.

